# Expected value of the Sackin index under biased speciation models

**DOI:** 10.1007/s00285-026-02465-4

**Published:** 2026-10-14

**Authors:** Daniel J. Bauman, Noah A. Rosenberg

**Affiliations:** 1 Institute for Computational and Mathematical Engineering, Stanford University, Stanford, CA 94305 USA; 2 Department of Biology, Stanford University, Stanford, CA 94305 USA

**Keywords:** Beta-splitting, Biased speciation model, Sackin index, Tree balance, Yule model

## Abstract

Models of random phylogenetic trees are important mathematical tools in phylogenetic data analysis; tree balance is a property of phylogenetic trees that helps describe and compare their shapes. Here, we investigate the Sackin index, one of the oldest and most widely used tree balance measures, under a variety of phylogenetic models beyond the standard Yule and Uniform models. The general biased speciation model is a biologically interpretable tree-generating model in which species branch into pairs of descendants according to an intrinsic branching rate, which is then split between the pair of descendants. The proportion of the branching rate of the parent that is assigned to a specific descendant, the split fraction, is a model parameter. Our main result is a formula for the expected value of the Sackin index under the general biased speciation model. Special cases of the general biased speciation model include a case that sets the split fraction to be a constant for each branching event, and two special cases inspired by the Aldous beta-splitting model: a random choice of split fraction is sampled independently at each branching event and drawn from a symmetric beta distribution in the Blum–François beta-splitting model or an asymmetric beta distribution in the Sainudiin–Véber beta-splitting model. As a corollary of our main result, we find formulas for the expected value of the Sackin index under these three cases of the general biased speciation model. We use the formulas to study how the balance of trees depends on the model parameters. Finally, because the Yule model is a special case of the Blum–François beta-splitting model, we find a new proof for the classical result describing the expected value of the Sackin index under the Yule model.

## Introduction

Phylogenetic trees are mathematical representations of hereditary structures in biology, such as the relationships between species that exist today and their common ancestors. In the setting of the descent of species, the shapes of phylogenetic trees contain information about the macroevolutionary forces that led to the relationships among those species (Kirkpatrick and Slatkin 1993; Mooers and Heard 1997; Stadler 2013; Morlon 2014). Many tree shape statistics that measure properties of trees, particularly the *tree balance indices*, have been introduced (Fischer et al. 2023). At the same time, numerous models for the generation of random trees have been proposed for exploring how assumptions about diversification rates and speciation patterns lead to different tree shapes (Heard 1996; Mooers et al. 2007; Lambert and Stadler 2013; Rosenberg et al. 2025). Intersecting these topics by examining tree shape statistics under various models of random trees provides predictions that can be used to assess the fit of empirical patterns in trees to macroevolutionary assumptions (Kersting et al. 2025).

The first proposed tree balance index, the Sackin index (Sackin 1972), examines the depths of the leaves of a tree. Although Sackin’s original proposal was to calculate the variance of the leaf depths (Coronado et al. 2020), the statistic commonly known as the *Sackin index* is instead defined by taking the *sum* of the leaf depths. The Sackin index remains one of the best-known tree balance indices (Fischer et al., 2023, p. 47). Its mathematical properties have been well studied under the two most widely used probability models for phylogenetic trees: the Yule or Yule–Harding model and the Uniform model (Fischer et al., 2023, ch. 5). The Uniform model assigns equal probability to each of the (2n-3)!! labeled trees with a given number of leaves *n*. The Yule model describes a branching process in which species split into new species at equal rates until arriving at *n* species in total. These two models widely serve as null models in phylogenetics. The distributions of phylogenetic tree properties under these models differ; for instance, the Uniform model generates trees that are more imbalanced on average than those generated by the Yule model (Steel, 2016, p. 50).

Studies that have compared the levels of balance in empirical reconstructed phylogenetic trees to those of the Yule and Uniform models have found that empirical trees do not match either model particularly well (Guyer and Slowinski 1991; Heard 1992; Mooers and Heard 1997; Aldous 2001; Blum and François 2006). Many alternative models have been subsequently proposed for generating phylogenetic trees (Morlon 2014; Kersting et al. 2025). For example, in 1996, Aldous introduced a family of probability distributions, the *Aldous beta-splitting model*, which can tune tree shapes from imbalanced to balanced according to a parameter $\beta \in (-2, \infty )$ (Aldous 1996, 2001). This family includes the Yule (β=0) and Uniform models (β=-3/2) as special cases. One property of the Aldous beta-splitting model is that it generates trees by starting from a current set of species and recursively joining them in clades to form the tree, a “backward-time” process, instead of by a “forward-time” process in which a single ancestor branches and diversifies to form the full tree (Aldous 1996; Sainudiin and Véber 2016). A forward-time model of constructing trees can be desirable because it possesses clearer evolutionary interpretability and it can encode biological information about the diversification rates of species.

Inspired in part by the Aldous beta-splitting model, Blum and François proposed a forward-time evolutionary model they termed a *biased speciation model* (Blum and François 2005, 2006). In this model, each existing species has some intrinsic diversification rate. When a split occurs, the two descendant species split the diversification rate *r* of the parent: they are assigned rates r(1-λ) and rλ for some parametric value of $\lambda \in [0,1]$, which we call the *split fraction* (Blum and François 2005, 2006). The original formulation, which we call the *biased speciation model with constant split fraction*, assumes that the split fraction λ is equal to the same constant across all splitting events.

To allow for a closer comparison with the Aldous beta-splitting model, Blum and François later considered a biased speciation model in which instead of being a parameter, the split fraction is a random variable *X*. In particular, each time a split occurs, the split fraction *X* is randomly sampled, independently from all other splits, from a beta distribution,

$$\begin{aligned} X \sim \text {Beta}(\alpha + 1, \alpha + 1), \end{aligned}$$

for a parameter $\alpha \in (-1, \infty )$ (Blum and François, 2006, eq. 7). This model, the *Blum–François beta-splitting model*, has a parameter α chosen to mimic the Aldous beta-splitting model: it generates highly imbalanced trees as $\alpha \rightarrow -1$, coincides with the Yule model if α=0 despite its quite different method for generating trees, and produces highly balanced trees as $\alpha \rightarrow \infty$  (Blum and François 2006).

Sainudiin and Véber later extended the Blum–François beta-splitting model by allowing the split fraction to follow an *asymmetric* beta distribution,

$$\begin{aligned} X \sim \text {Beta}(\alpha + 1, \beta + 1), \end{aligned}$$

for $\alpha , \beta \in (-1, \infty )$ (Sainudiin and Véber 2016). Allowing asymmetries in the speciation rates of descendant species can be a natural model choice; for instance, a speciation mechanism that assumes that the two descendant species of a parent are the parent itself and a new species that split from the parent would likely have such asymmetries (Sainudiin and Véber 2016). We call this model the *Sainudiin–Véber beta-splitting model*.

**Fig. 1:**
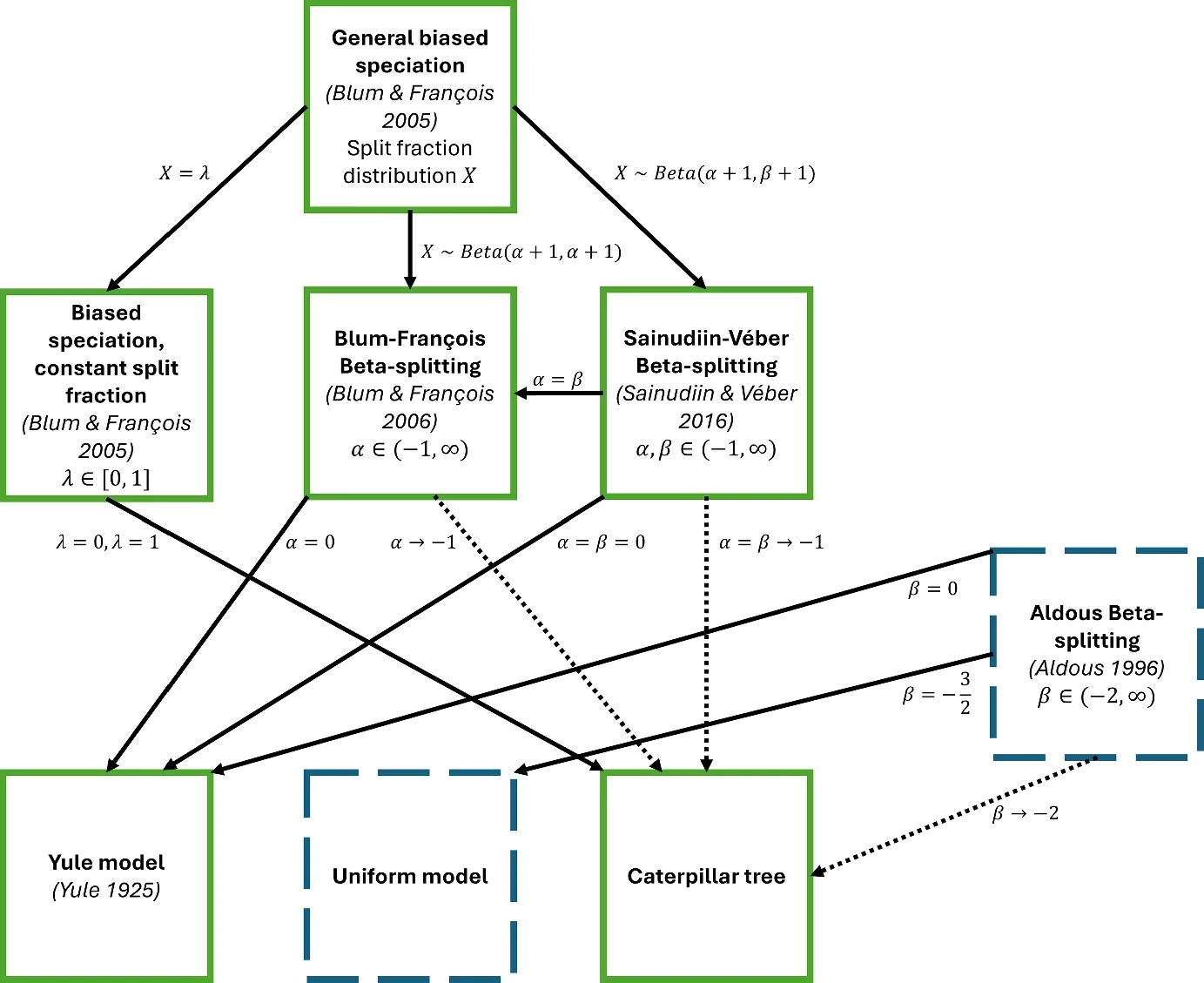
Relationships between the general biased speciation model and other models of randomly generated phylogenetic trees. Models colored in green (solid boxes) have a simple biological interpretation as a forward-time branching process with fixed branching rates for each species, whereas those colored in blue (dashed boxes) do not. An arrow from model A to model B indicates that B is a special case of A that emerges from a particular choice of parameters; a dotted arrow indicates that the special case is a limiting value of the parameter. Classical results about the Sackin index for the three bottom models, the Yule model, Uniform model, and caterpillar tree, are summarized in Theorem 1. We prove a result about the Sackin index under the general biased speciation model in Theorem  2. Subsequent examination of its three special cases, the biased speciation model with constant split fraction (X = λ), the Blum–François beta-splitting model, and the Sainudiin–Véber beta-splitting model, appear in Sects. 4.1, 4.2, and 4.3, respectively. For a reference for the caterpillar tree, see Steel (Steel, 2016, p. 11), and for the Uniform model, see Steel  (Steel, 2016, p. 50) (color figure online)

The biased speciation model with constant split fraction (i.e. X=λ), Blum–François beta-splitting model, and Sainudiin–Véber beta-splitting model are three special cases of a model that we call the *general biased speciation model*. We use this term to describe any tree-generating model that assumes that each extant species has a constant diversification rate and that splits the diversification rate of a parent species between its immediate descendants. Each clade in the tree has a diversification rate corresponding to the sum of the diversification rates of all leaves in the clade. The method of assigning diversification rates to descendants of an internal node ensures that as the tree-generating process continues and more leaves are added to the tree, the total diversification rate of a clade remains constant. This method differs from the random process used to generate trees under the Yule model, for instance. In that process, when more leaves are added to a single clade, the total diversification rate of that clade increases with the number of leaves in the clade. Therefore, the key biological assumption of the general biased speciation model is that the evolution of the tree is driven by each clade possessing a constant rate of generating new species relative to all other clades. The choice of the distribution for the split fraction *X* tunes the model between trees that are highly imbalanced and trees that are highly balanced, as in Aldous’s beta-splitting model. In Fig. 1, we display the relationships between the various stochastic phylogenetic models discussed above, along with the maximally imbalanced tree, the caterpillar tree.

The central result of our paper is a derivation of the expected value of the Sackin index under the general biased speciation model. In Sect. 2, we formally describe the general biased speciation model and introduce notation and definitions. In Sect. 3, we describe relevant known results about the Sackin index and state our main theorem. The proof of the theorem is left to Appendix A. In Sect. 4, we study the formulas recovered from applying our theorem to three special cases of the general biased speciation model: the biased speciation model with constant split fraction (X=λ), the Blum–François beta-splitting model, and the Sainudiin–Véber beta-splitting model. We conclude with a discussion of the results and potential applications to future problems.

## Definitions and preliminaries

After describing notation in Sect. 2.1, we introduce the general biased speciation model in Sect. 2.2.

### Definitions

A (nonempty) *rooted binary tree* $T = \big (V(T), E(T)\big )$ with vertex set *V*(*T*) and an edge set *E*(*T*) is a directed acyclic graph with the following properties: (i) vertices have in-degree 0 or 1; (ii) there exists exactly one vertex $\rho \in V(T)$, called the *root* of the tree, with in-degree 0; (iii) each vertex has out-degree 0 (*leaves*) or 2 (*internal vertices*). Henceforth, the term *phylogenetic tree* or simply *tree* refers to a rooted, binary, nonempty tree.

We consider trees in which vertices are unlabeled. Two trees $T_1, T_2$ are equivalent if they have the same shape; that is, we consider the unlabeled rooted binary tree shapes enumerated by the Wedderburn–Etherington numbers, the first of which are 1,1,1,2,3,6,11,23,46,98… for n=1 to 10 leaves.

The set of leaves of *T* is denoted $V_L(T)$. The set of internal vertices is denoted $\mathring{V}(T) = V(T) \setminus V_L(T)$. The *size* of a tree is its number of leaves: $|T| = |V_L(T)|$. The set of rooted binary unlabeled trees with *n* leaves is denoted $\mathcal {T}_n$. We denote the set of all nonempty trees by $\mathcal {T} = \cup _{n=1}^\infty \mathcal {T}_n$.

The direction of the edges of a tree is outward from the root. If a path exists from vertex *u* to vertex *v*, then we say that *v* is *descended* from *u*, and *u* is an *ancestor* of *v*; if this path is nontrivial (u≠v) and it contains exactly one edge, then *v* is a *child* of *u*, and *u* is the *parent* of *v*.

We will have occasion to consider special types of trees. A *cherry* is a tree that has three nodes: the root and two leaves. A *caterpillar* is a tree that has exactly one cherry.

For all vertices v∈V(T), there exists a unique directed path from ρ to *v*. The *depth* of a vertex *v*, $\delta _T(v)$, is the number of edges separating ρ and *v* in this path. The *Sackin index* *S*(*T*) of a tree *T* is defined by

1 $$\begin{aligned} S(T) = \sum _{v \in V_L(T)} \delta _T(v). \end{aligned}$$

An *unlabeled history* is a sequence of trees $\{T_1, T_2, \ldots , T_n\}$ such that (i) for each *k*, $1 \le k \le n$, $T_k \in \mathcal {T}_k$, and (ii) for each *k*, $1 \le k \le n-1$, the tree $T_{k+1}$ can be formed by replacing one of the leaves of $T_k$ by a cherry. An unlabeled history can be regarded as representing the sequence of splitting events that transform a single ancestor species into a set of *n* unlabeled species with phylogenetic relationships represented by $T_n$.

### General biased speciation model

We consider the *general biased speciation model*, a process that generates a random unlabeled history. This model takes one input, the *split fraction* *X*, a random variable with cumulative distribution function $F(x) = \mathbb {P}[X \le x]$. The only requirement of this c.d.f. is that the random variable *X* must take values in the range [0, 1], or $\lim _{x \rightarrow 0^-} F(x) = 0$ and F(1)=1. The distribution of *X* can arise from a parametrized family of probability distributions, in which case we consider the parameters defining the c.d.f. *F*(*x*) to be the parameters of the model.

Fix n≥1. The model generates an unlabeled history $\{T_1, T_2, \ldots , T_n\}$ as follows. At time k=1, begin with the unlabeled history $\{T_1\}$ containing a single tree with one vertex. This vertex ρ is assigned a split probability $p_{\rho } = 1$. Let $1 \le k \le n-1$, and assume the current unlabeled history $\{T_1, T_2, \ldots , T_k\}$ generated by the model has the property that each leaf $v \in V_L(T_k)$ is assigned a split probability $p_v \ge 0$ such that

2 $$\begin{aligned} \sum _{v \in V_L(T_k)} p_v = 1. \end{aligned}$$

To generate $T_{k+1}$ from $T_k$, one leaf $w \in V_L(T_k)$ is randomly selected according to the split probabilities of the leaves, a valid probability distribution by Eq. 2. This leaf $w \in V_L(T_k)$ is replaced by a cherry to form a new tree $T_{k+1}$, which is appended to the unlabeled history. The split probability of each node $v \in V_L(T_k) \setminus \{w\}$ that did not split at time *k* remains unchanged in $T_{k+1}$. The two descendants of *w* in $T_{k+1}$ are assigned split probabilities $p_w X_{k+1}$ and $p_w (1 - X_{k+1})$, where $\{X_{k}\}_{k=2}^n$ is a collection of independent and identically distributed random variables with the same distribution as *X*. Because $p_w(1 - X_{k+1}) + p_w X_{k+1} = p_w$, the total split probability of all leaves in $T_{k+1}$ remains $\sum _{v \in V_L(T_{k+1})} p_v = 1$.

**Fig. 2:**
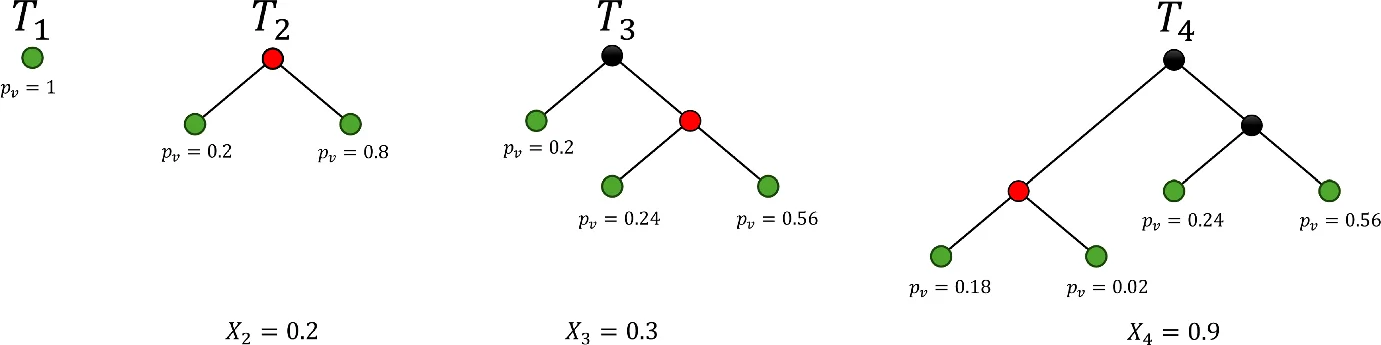
Example of the general biased speciation model generating a sequence of trees $\{T_1, T_2, T_3, T_4\}$. The initial tree $T_1$ is a tree with one node with split probability 1. At times k=2,3,4, a random leaf *w* from $T_{k-1}$ (shown in red) is chosen to split into a cherry according to the split probabilities $p_v$ of the leaves. The descendants of the chosen node receive split probabilities equal to $X_k p_w$ and $(1 - X_k) p_w$, where $X_k\sim X$ are variates sampled i.i.d. from the split fraction distribution *X* (color figure online)

By performing this process of generating split probabilities at times k=1,2…,n-1, an unlabeled history $\{T_1, T_2, \ldots , T_n\}$ is generated (Fig. 2). The probability of reaching a given final tree $T_n$ follows the probability distribution on $\mathcal {T}_n$ induced by the model. The choice of split fraction describes which biased speciation model is used; if *X* is specified, then we refer to the model as the *biased speciation model with split fraction* *X*. Because the two descendants of a parent node are indistinguishable, the model with split fraction *X* is identical to the model with split fraction 1-X. We consider three choices for *X*:

1. The *biased speciation model with constant split fraction* is the biased speciation model whose split fraction *X* follows a trivial distribution, $\mathbb {P}[X = \lambda ] = 1$ for a parameter $\lambda \in [0,1]$ (Blum and François 2005).
2. The *Blum–François beta-splitting model* is the biased speciation model with split fraction $X \sim \text {Beta}(\alpha + 1, \alpha + 1)$ for a parameter $\alpha \in (-1, \infty )$ (Blum and François 2006).
3. The *Sainudiin–Véber beta-splitting model* is the biased speciation model with split fraction $X \sim \text {Beta}(\alpha + 1, \beta + 1)$ for parameters $\alpha , \beta \in (-1, \infty )$ (Sainudiin and Véber 2016).

## Expected Sackin index in the general biased speciation model

We describe known results concerning the Sackin index in Theorem  1. This theorem compiles several results summarized by (Fischer et al., 2023, chapter 5).

### Theorem 1

(Fischer et al. (2023), chapter 5) The Sackin index has the following properties:

1. Among all trees $T \in \mathcal {T}_n$, the maximal value of the Sackin index, which we label $S_{\max }(n)$, is
  3 $$\begin{aligned} S(T) \le \frac{n^2 + n - 2}{2}. \end{aligned}$$
  $S(T)=S_{\max }(n)$ if and only if *T* is the caterpillar tree of size *n*.
2. Among all trees $T \in \mathcal {T}_n$, the minimal value of the Sackin index, which we label $S_{\min }(n)$, is
  4 $$\begin{aligned} S(T) \ge n (r + 3) - 2^{r + 2}, \end{aligned}$$
  where $r = \lfloor \log _2 n \rfloor - 1$. For all n≥1, there exists at least one tree *T* of size *n* with $S(T) = S_{\min }(n)$.
3. Consider a phylogenetic tree $T \in \mathcal {T}_n$ sampled under the Yule model. The expected value of the Sackin index, which we label *Y*(*n*), satisfies
  5 $$\begin{aligned} \mathbb {E}_Y\big [S(T) \big ] = 2n \, (H_{n} - 1), \end{aligned}$$
  where $H_n = \sum _{i=1}^n {1}/{i}$ denotes the harmonic number.
4. Consider a phylogenetic tree $T \in \mathcal {T}_n$ sampled under the Uniform model. The expected value of the Sackin index, which we label *U*(*n*), satisfies
  6 $$\begin{aligned} \mathbb {E}_U \big [ S(T) \big ] = \frac{n! \, (n-1)!}{(2n-2)!} \bigg [ 2^{2n-2} - \left( {\begin{array}{c}2n-2\\ n-1\end{array}}\right) \bigg ]. \end{aligned}$$

Part 1 of Theorem 1 indicates that across trees with *n* leaves, the Sackin index is maximized by highly imbalanced caterpillar trees. Trees that achieve the minimum in part 2 are highly balanced; note that the minimum is attained by multiple trees unless $n \in \{2^m - 1, 2^m, 2^m + 1\}$ for some m≥1 (Fischer, 2021, Corollary 1). Parts 3 and 4 of Theorem 1 give the expected value of the Sackin index under the Yule and Uniform models. We now state our main theorem, which generalizes these expectations to the general biased speciation model.

### Theorem 2

Let $\{T_1, T_2, \ldots , T_n\}$ be a random sequence of trees generated under the general biased speciation model with split fraction *X*. The expected value of the Sackin index $S(T_n)$ is

7 $$\begin{aligned} \mathbb {E}\big [S(T_n)\big ] = \frac{n^2 + n - 2}{2} + \sum _{k=3}^{n-1}\left( {\begin{array}{c}n-1\\ k\end{array}}\right) \prod _{j=2}^{k-1} e_j, \end{aligned}$$

where $e_j$ is a quantity that depends on the moments of *X*, satisfying

8 $$\begin{aligned} e_j = \mathbb {E}\big [X^j \big ] + \mathbb {E}\big [(1-X)^j \big ] - 1. \end{aligned}$$

Observe that the first term in Eq. 7 is equal to the maximal value of the Sackin index, achieved by the caterpillar tree (Eq. 3). The summation term can be interpreted as an adjustment arising from the specific choice of split fraction *X* in the model. Because the expectation of a random variable can be no larger than its maximal value, the summation in Eq. 7 is non-positive.

The proof of Theorem 2 appears in Appendix A. Briefly, the proof studies how the Sackin index changes over time as the model generates a random unlabeled history $\{T_1, T_2, \ldots , T_n\}$. A recursive equation then studies the conditional expectation of the Sackin index in a single time step in the model (Sect. A.2). Because the Sackin index measures only the tree topology but the biased speciation model also involves the split probabilities of the leaves of the tree, we must track auxiliary statistics that examine the single-step change in properties of the split probabilities. A technical lemma (Sect. A.3) allows us to recursively solve a system of linear equations to recover the expected value of the Sackin index under the model (Sect. A.4), proving Theorem 2.

## Expected Sackin index in special cases of the general biased speciation model

We next study three special cases of Theorem 2, corresponding to different choices for the split fraction *X*: the biased speciation model with constant split fraction (Sect. 4.1), the Blum–François beta-splitting model (Sect. 4.2), and the Sainudiin–Véber beta-splitting model (Sect. 4.3). For each special case, we find the formula given by eq. 7 and evaluate it analytically and numerically. We study the balance of trees with different parameter choices in relation to the values in Theorem 1 for $S_{\min }(n)$ and $S_{\max }(n)$, the minimum and maximum values of the Sackin index, and *Y*(*n*) and *U*(*n*), the expected value of the Sackin index under the Yule and Uniform models, respectively.

For our numerical evaluations of Eq. 7, we used high-precision arithmetic as implemented using Python’s mpmath package, version 1.3.0 (The mpmath development team 2023). Using a decimal precision with $\frac{n}{2}$ digits sufficed to stably calculate eq. 7 in each special case, which we tested up to n=50000. All code to numerically compute the equation and to reproduce the figures can be found at https://github.com/dbauman18/biased_speciation_calculation.

### Biased speciation model with constant split fraction

The biased speciation model with constant split fraction assumes that the split fraction is a constant, X=λ with probability 1 (Blum and François 2005, 2006). Although the parameter λ can take values $\lambda \in [0,1]$, without loss of generality we can assume $\lambda \in [0, \frac{1}{2}]$ because we do not distinguish between the two children of an internal node. By applying Theorem 2 to this model, we obtain the following result.

#### Corollary 3

Let $\{T_1, T_2, \ldots , T_n\}$ be a random sequence of trees generated under the biased speciation model with constant split fraction with parameter $\lambda \in [0, \frac{1}{2}]$. The expected value of the Sackin index $S(T_n)$, which we label F(n,λ), satisfies

9 $$\begin{aligned} \mathbb {E}\big [ S(T_n) \big ] = \frac{n^2 + n - 2}{2} + \sum _{k=3}^{n-1}\left( {\begin{array}{c}n-1\\ k\end{array}}\right) \prod _{j=2}^{k-1} \big [\lambda ^j + (1 - \lambda )^j- 1\big ]. \end{aligned}$$

#### Proof

The result follows from Theorem 2, noting that if *X* is constant, X=λ, then the quantity $e_j$ simplifies to $\lambda ^j + (1-\lambda )^j - 1$. □

The case λ=0 represents a model that produces caterpillar trees with probability 1. Accordingly, with λ=0, the product vanishes and we recover the Sackin index of the caterpillar tree,

10 $$\begin{aligned} F(n,0) = \frac{n^2 + n - 2}{2} = S_{\max }(n). \end{aligned}$$

**Fig. 3:**
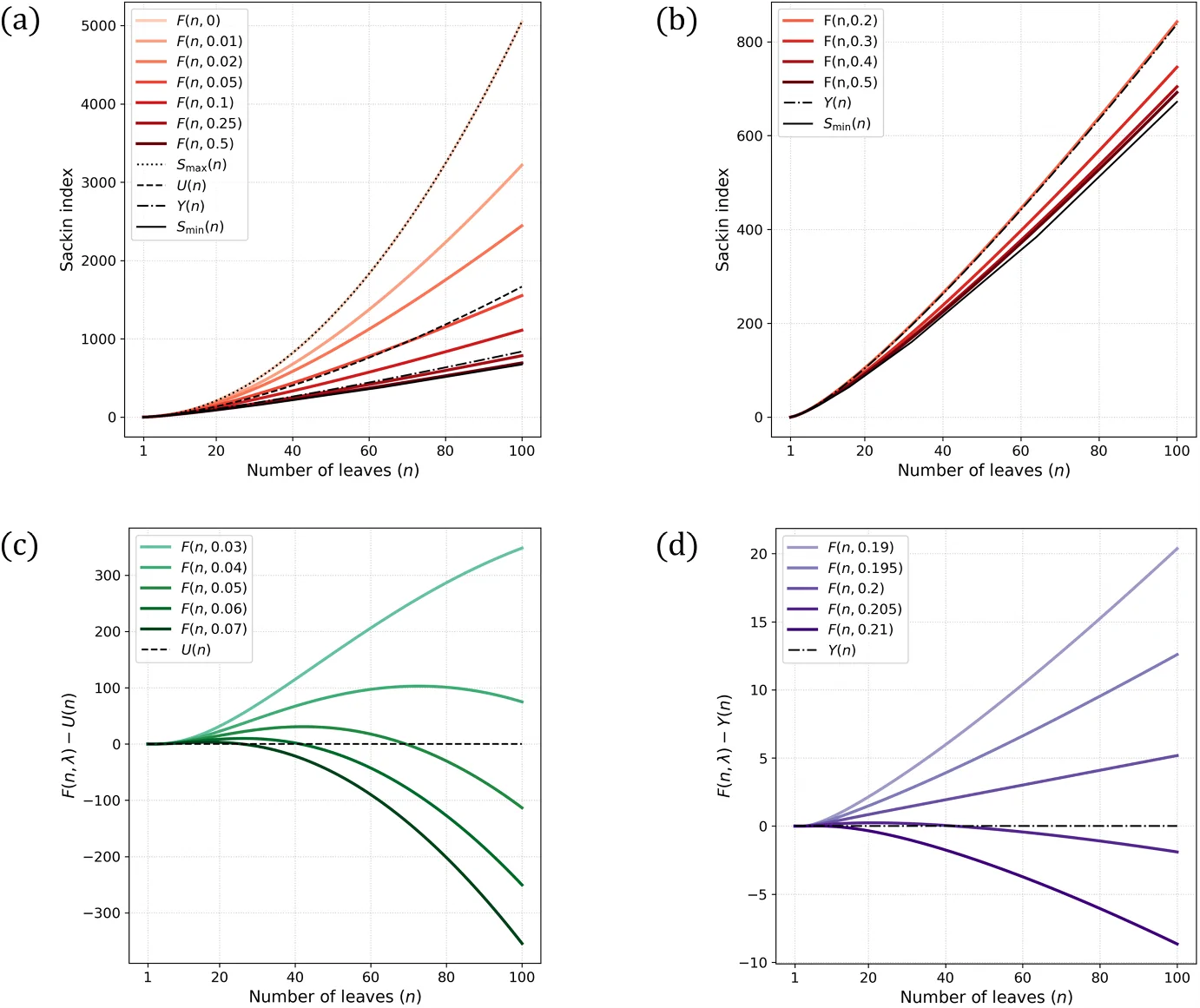
Expected value of the Sackin index under the biased speciation model with constant split fraction. For $1 \le n \le 100$, F(n,λ) (Eq. 9), the expected value of the Sackin index under the biased speciation model with constant split fraction λ, is plotted in comparison to the reference values of the Sackin index given by Theorem 1: $S_{\max }(n)$ (Eq. 3), the maximum value; $S_{\min }(n)$ (Eq. 4), the minimum value; *U*(*n*) (Eq. 6), the expected value under the Uniform model; and *Y*(*n*) (Eq. 5), the expected value under the Yule model. **a** F(n,λ) for λ in [0, 0.5]. **b** F(n,λ) for a narrower range of λ, in [0.2, 0.5]. **c** F(n,λ)-U(n) for values of λ for which F(n,λ) is near *U*(*n*). **d** F(n,λ)-Y(n) for values of λ for which F(n,λ) is near *Y*(*n*)

We numerically evaluated F(n,λ) for $1 \le n \le 100$ and a variety of values of $\lambda \in [0, \frac{1}{2}]$. We observe in Fig. 3a that if λ is fixed, then F(n,λ) increases monotonically in *n*. If, on the other hand, *n* is fixed, then F(n,λ) decreases monotonically for $\lambda \in [0, \frac{1}{2}]$. We observe numerically that at its minimal value of $\lambda = \frac{1}{2}$, F(n,λ) nears but does not reach $S_{\min }(n)$ (Eq. 4), as shown in Fig. 3b.

We further compared F(n,λ) to *U*(*n*) and *Y*(*n*), the expected values of the Sackin index under the Uniform and Yule models. Noting that in Fig. 3a, *F*(*n*, 0.05) is close to *U*(*n*), in Fig. 3c, we plot F(n,λ)-U(n) for several values of λ near 0.05. Also noting that in Fig. 3b, *F*(*n*, 0.2) is close to *Y*(*n*), in Fig. 3d, we plot F(n,λ)-Y(n) for several values of λ near 0.2.

We observe in Fig. 3c that although F(n,λ) comes close to approximating the reference model mean *U*(*n*) for some specific values of λ, no value of λ provides an exact fit across a range of values of *n*, indicating that no parameter choice in this model generates the exact Uniform distribution of trees. Similarly, in Fig. 3d, no value of λ provides an exact fit of F(n,λ) to *Y*(*n*), indicating that no parameter choice generates the exact Yule distribution. The closer fit in Fig. 3d suggests that *Y*(*n*) can be approximated by F(n,λ) more closely than can *U*(*n*) for an appropriate choice of λ.

### Blum–François beta-splitting model

Next, we examine the Blum–François beta-splitting model, the biased speciation model with split fraction $X \sim \text {Beta}(\alpha + 1, \alpha + 1)$ for $\alpha \in (-1, \infty )$. Note that Blum and François referred to their model as “beta-binomial”; we call it “beta-splitting” as in the later usage by Sainudiin and Véber (2016). Theorem 2 gives the following result.

#### Corollary 4

Let $\{T_1, T_2, \ldots , T_n\}$ be a random sequence of trees generated under the Blum–François beta-splitting model with parameter $\alpha \in (-1, \infty )$. The expected value of the Sackin index $S(T_n)$, which we label G(n,α), satisfies

11 $$\begin{aligned} \mathbb {E}\big [ S(T_n) \big ] = \frac{n^2 + n - 2}{2} + \sum _{k=3}^{n-1}\left( {\begin{array}{c}n-1\\ k\end{array}}\right) \prod _{j=2}^{k-1} \bigg [2\frac{\Gamma (\alpha + 1 + j) \, \Gamma (2 \alpha + 2)}{ \Gamma (2 \alpha + 2 + j) \, \Gamma (\alpha + 1)} - 1 \bigg ]. \end{aligned}$$

#### Proof

For a $\text {Beta}(a,b)$ random variable *X*, we use the standard result (Gupta and Nadarajah, 2004, p. 35)

12 $$\begin{aligned} \mathbb {E}\big [X^j\big ]&= \frac{\Gamma (a+b) \, \Gamma (a+j)}{\Gamma (a+b+j) \, \Gamma (a)}. \end{aligned}$$

Because the variable 1-X satisfies (Gupta and Nadarajah, 2004, p. 33)

13 $$\begin{aligned} 1-X \sim \text {Beta}(b, a), \end{aligned}$$

Eqs. 12 and 13 together imply that

14 $$\begin{aligned} \mathbb {E}\big [(1-X)^j\big ]&= \frac{\Gamma (a+b) \, \Gamma (b+j)}{\Gamma (a+b+j) \, \Gamma (b)}. \end{aligned}$$

Using α+1 in the roles of both *a* and *b* in Eqs. 12 and 14, the value of $e_j$ from Eq. 8 is given by

15 $$\begin{aligned} e_j = \mathbb {E}\big [X^j \big ] + \mathbb {E}\big [(1-X)^j \big ] - 1 = 2\frac{\Gamma (\alpha + 1 + j) \, \Gamma (2 \alpha + 2)}{ \Gamma (2 \alpha + 2 + j) \, \Gamma (\alpha + 1)} - 1. \end{aligned}$$

The result then follows from Theorem 2. □

If α=0, then the Blum–François beta-splitting model generates the same distribution of trees as the Yule model (Blum and François 2006), which implies that

16 $$\begin{aligned} G(n, 0) = Y(n). \end{aligned}$$

Substituting α=0 in Eq. 11, we simplify the resulting formula to verify Eq. 16, producing a new proof for the expected value of the Sackin index under the Yule model; see Appendix B for details.

Additionally, we can interpret the limits $\alpha \rightarrow -1^+$ and $\alpha \rightarrow \infty$. For each limit, it suffices to evaluate the limit of $e_j$ in Eq. 15.

We claim that $e_j \rightarrow 0$ as $\alpha \rightarrow -1^+$ for all j≥2, which implies that

17 $$\begin{aligned} \lim _{\alpha \rightarrow -1^+} G(n,\alpha ) = F(n, 0) = S_{\max }(n). \end{aligned}$$

To verify the claim, we evaluate Eq. 15 with $\alpha \rightarrow -1^+$ using the identity (Olver et al., 2010, eq. 5.5.1)

18 $$\begin{aligned} \Gamma (z+1) = z \Gamma (z), \end{aligned}$$

for $z \ne 0, -1, -2, -3, \ldots$. At α=-1, $\Gamma (\alpha +1+j)/\Gamma (2\alpha +2+j) = 1$. For α>-1, we can write $\Gamma (2\alpha +2)/\Gamma (\alpha +1)=(\alpha +1)\Gamma (2\alpha +3)/[(2\alpha +2)\Gamma (\alpha +2)] = \frac{1}{2} \Gamma (2\alpha +3)/\Gamma (\alpha +2)$, from which $\lim _{\alpha \rightarrow -1^+} [\Gamma (2\alpha +2)/\Gamma (\alpha +1)] = \frac{1}{2}$ and $e_j \rightarrow 0$, verifying Eq. 17.

For the $\alpha \rightarrow \infty$ limit, recall that as $x \rightarrow \infty$, we have an asymptotic result for the ratio of two gamma functions (Olver et al., 2010, eq. 5.11.12):

19 $$\begin{aligned} \frac{\Gamma (x + a)}{\Gamma (x + b)} \sim x^{a-b}. \end{aligned}$$

Consequently, as $$\alpha \rightarrow \infty $$,

20 $$\begin{aligned} \frac{\Gamma (\alpha + 1 + j) \, \Gamma (2\alpha + 2)}{\Gamma (2\alpha + 2 + j) \, \Gamma (\alpha + 1)} = \frac{\Gamma (\alpha + 1 + j)}{\Gamma (\alpha + 1)} \, \frac{\Gamma (2\alpha + 2)}{\Gamma (2\alpha + 2 + j)} \sim \alpha ^j (2\alpha )^{-j} = 2^{-j}. \end{aligned}$$

Plugging this limit into Eq. 15, we observe that the limit directly matches the $\lambda = \frac{1}{2}$ case of Eq. 11, the result from Sect. 4.1 for the model with constant split fraction. In other words,

$$\begin{aligned} \lim _{\alpha \rightarrow \infty } G(n,\alpha ) = F\Big (n, \frac{1}{2}\Big ). \end{aligned}$$

**Fig. 4:**
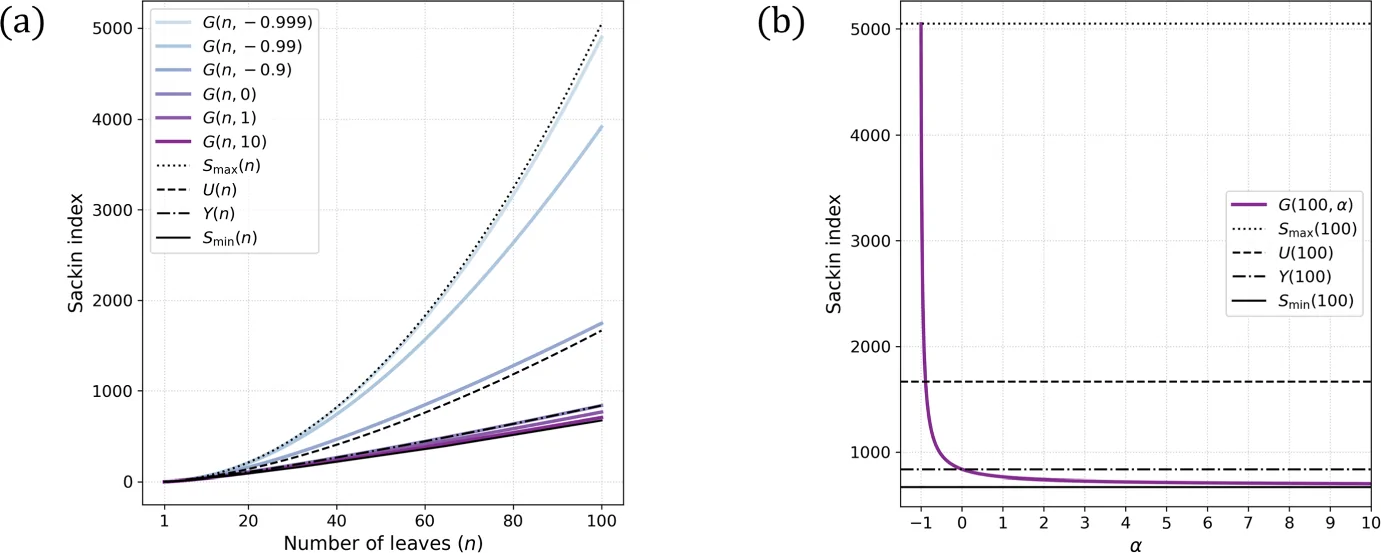
Expected value of the Sackin index under the Blum–François beta-splitting model. For $1 \le n \le 100$, G(n,α) (Eq. 11), the expected value of the Sackin index under the Blum–François beta-splitting model, is plotted in comparison to the reference values of the Sackin index given by Theorem 1: $S_{\max }(n)$ (Eq. 3), the maximum value; $S_{\min }(n)$ (Eq. 4), the minimum value; *U*(*n*) (Eq. 6), the expected value under the Uniform model; and *Y*(*n*) (Eq. 5), the expected value under the Yule model. (a) G(n,α) for $1 \le n \le 100$ and specific values of α in (-1,10]. (b) G(n,α) at n=100 for $\alpha \in (-1, 10]$

For several values of $\alpha \in (-1, \infty )$, we numerically evaluated G(n,α). We observe in Fig. 4a that for a fixed α, G(n,α) increases monotonically in *n*. Figure 4b demonstrates that for a fixed *n*, G(n,α) decreases monotonically in α as α increases from -1 toward ∞. Both plots show that if α=0, then G(n,α)=Y(n), which agrees with the theoretical result that this model is identical to the Yule model for α=0.

### Sainudiin–Véber beta-splitting model

Relaxing the assumption that the beta distribution is symmetric and considering the biased speciation model with split fraction $X \sim \text {Beta}(\alpha + 1, \beta + 1)$ for $\alpha , \beta \in (-1, \infty )$, we arrive at the Sainudiin–Véber beta-splitting model (Sainudiin and Véber 2016). Note that although the model has two parameters, alpha and beta, we continue to use the term “beta-splitting,” treating “beta” in “beta-splitting” as a reference to the use of a beta distribution, rather than to the parameter β.

By applying Theorem 2, we obtain the following result.

#### Corollary 5

Let $\{T_1, T_2, \ldots , T_n\}$ be a random sequence of trees generated under the Sainudiin–Véber beta-splitting model with parameters $\alpha , \beta \in (-1, \infty )$. The expected value of the Sackin index $S(T_n)$, which we label $H(n, \alpha , \beta )$, satisfies

21 $$\begin{aligned} \mathbb {E}\big [ S(T_n) \big ] &  = \frac{n^2 + n - 2}{2} + \sum _{k=3}^{n-1}\left( {\begin{array}{c}n-1\\ k\end{array}}\right) \prod _{j=2}^{k-1} \bigg [\frac{\Gamma (\alpha + 1 + j) \, \Gamma (\alpha + \beta + 2)}{\Gamma (\alpha + \beta + 2 + j) \, \Gamma (\alpha + 1)} \nonumber \\ &  \qquad + \frac{\Gamma (\beta + 1 + j) \, \Gamma (\alpha + \beta + 2)}{\Gamma (\alpha + \beta + 2 + j) \, \Gamma (\beta + 1)} - 1 \bigg ]. \end{aligned}$$

#### Proof

Applying Eqs. 12 and 14, the value of $e_j$ from Eq. 8 is given by

22 $$\begin{aligned} e_j = \frac{\Gamma (\alpha + 1 + j) \, \Gamma (\alpha + \beta + 2)}{\Gamma (\alpha + \beta + 2 + j) \, \Gamma (\alpha + 1)} + \frac{\Gamma (\beta + 1 + j) \, \Gamma (\alpha + \beta + 2)}{\Gamma (\alpha + \beta + 2 + j) \, \Gamma (\beta + 1)} - 1. \end{aligned}$$

The result then follows immediately from Theorem 2. □

If $\alpha = \beta$, then we recover the Blum–François beta-splitting model from Sect. 4.2, and

$$\begin{aligned} H(n, \alpha , \alpha ) = G(n,\alpha ). \end{aligned}$$

Switching α and β in Eq. 22 does not change the expression for $e_j$, so *H* has a symmetry:

23 $$\begin{aligned} H(n,\alpha , \beta ) = H(n, \beta , \alpha ). \end{aligned}$$

We can examine various limiting cases. First, fix $\alpha \in (-1, \infty )$. As $\beta \rightarrow -1^+$, we use Eq. 18 to write $\Gamma (\beta +1 +j) / \Gamma (\beta + 1) = (\beta + 1) \, \Gamma (\beta + 2 + j) / [(\beta + 1 + j) \, \Gamma (\beta + 2)]$, which implies $\lim _{\beta \rightarrow -1^+} \Gamma (\beta +1 +j) / \Gamma (\beta + 1) = 0$ and hence $e_j \rightarrow 0$ in Eq. 22.

Similarly, with $\alpha \in (-1, \infty )$ fixed and $\beta \rightarrow \infty$, we use Eq. 19 to find that

24 $$\begin{aligned} \frac{\Gamma (\alpha + 1 + j) \, \Gamma (\alpha + \beta + 2)}{\Gamma (\alpha + \beta + 2 + j) \, \Gamma (\alpha + 1)} \sim \frac{\Gamma (\alpha + 1 + j)}{\Gamma (\alpha + 1)} \, \beta ^{-j} \rightarrow 0. \end{aligned}$$

With $\alpha \in (-1, \infty )$ fixed and $\beta \rightarrow \infty$,

25 $$\begin{aligned} \frac{\Gamma (\beta + 1 + j) \, \Gamma (\alpha + \beta + 2)}{\Gamma (\alpha + \beta + 2 + j) \, \Gamma (\beta + 1)} \sim \beta ^j \cdot \beta ^{-j} = 1. \end{aligned}$$

Substituting Eqs. 24 and 25 into Eq. 22, we conclude $e_j \rightarrow 0$ in this case.

We have shown that

26 $$\begin{aligned} \lim _{\beta \rightarrow -1^+} H(n, \alpha , \beta ) = \lim _{\beta \rightarrow \infty } H(n, \alpha , \beta ) = F(n, 0) = S_{\max }(n). \end{aligned}$$

Combining Eq. 26 with Eq. 23 leads to

$$\begin{aligned} \lim _{\alpha \rightarrow -1^+} H(n, \alpha , \beta ) = \lim _{\alpha \rightarrow \infty } H(n, \alpha , \beta ) = F(n, 0) = S_{\max }(n). \end{aligned}$$

More generally, fix a value of λ in $(0,\frac{1}{2}]$. Consider the limit in which $\alpha , \beta \rightarrow \infty$ while fixing the ratio $\frac{\alpha }{\beta }$ at $\frac{\lambda }{1-\lambda }$. Applying Eq. 19 and Eq. 22 in this limit yields

27 $$\begin{aligned} &  \frac{\Gamma (\alpha + 1 + j) \, \Gamma (\alpha + \beta + 2)}{\Gamma (\alpha + \beta + 2 + j) \, \Gamma (\alpha + 1)} + \frac{\Gamma (\beta + 1 + j) \, \Gamma (\alpha + \beta + 2)}{\Gamma (\alpha + \beta + 2 + j) \, \Gamma (\beta + 1)} \nonumber \\ &  \qquad \sim \alpha ^j (\alpha + \beta )^{-j}+ \beta ^j (\alpha + \beta )^{-j} = \lambda ^j + (1-\lambda )^j. \end{aligned}$$

By substituting into Eq. 21, we observe agreement with the model with constant split fraction from Sect. 4.1 with parameter λ, or

$$\begin{aligned} \lim _{\begin{array}{c} \alpha , \beta \rightarrow \infty \\ {\alpha /\beta = \lambda /(1-\lambda )} \end{array}} H(n, \alpha , \beta ) = F(n,\lambda ). \end{aligned}$$

**Fig. 5:**
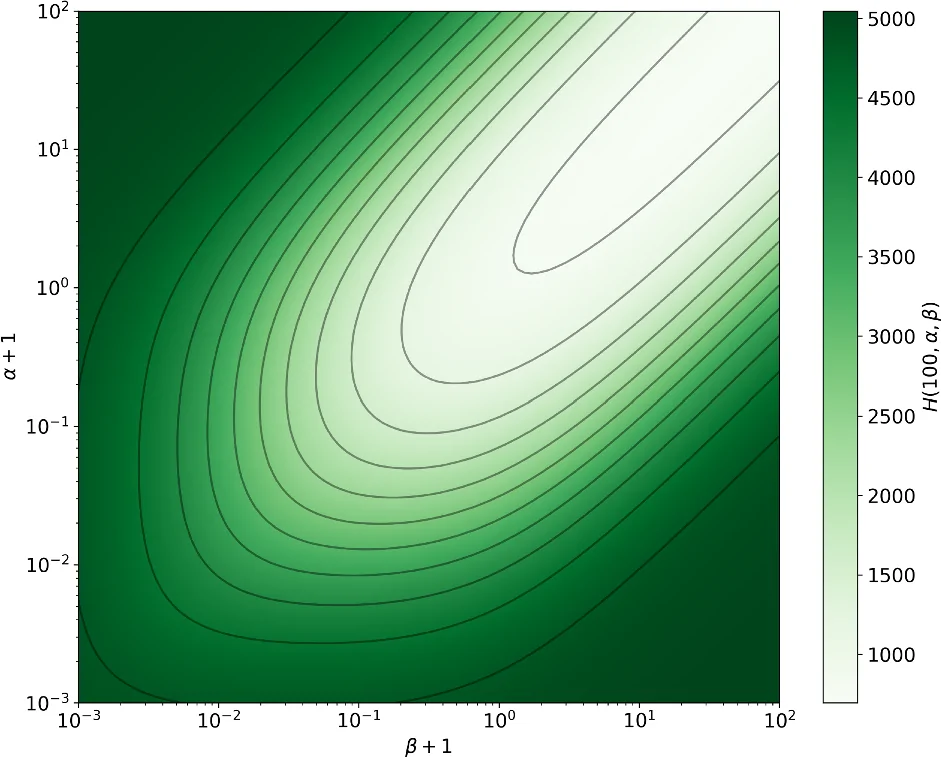
Expected value of the Sackin index under the Sainudiin–Véber beta splitting model. For n=100, $H(n, \alpha , \beta )$ (Eq. 21), the expected value of the Sackin index under the Sainudiin-Véber beta splitting model, is plotted. Values of the function are calculated over a 100×100 grid of logarithmically spaced points for $(\alpha + 1, \beta + 1) \in \big [10^{-3}, 10^2\big ] \times \big [10^{-3}, 10^2\big ]$. Contours indicate curves in parameter space that have the same value of $H(n, \alpha , \beta )$

In Fig. 5, we plot $H(n, \alpha , \beta )$ for a fixed n=100 as a function of the parameters α and β (shifted to $(\alpha + 1, \beta + 1)$ for ease of displaying the logarithmically scaled axes). When α is fixed and β is varied, observe that $H(n, \alpha , \beta )$ is not monotonic: it is maximal at its endpoints $\beta \rightarrow -1^+$ and $\beta \rightarrow \infty$ by Eq. 26, and the function reaches some minimum value at a location between these endpoints.

## Discussion

We have computed the expected value of the Sackin index under the general biased speciation model. As a corollary to our main Theorem 2, we have obtained exact formulas for the expected value of the Sackin index in three special cases involving previously studied models: the biased speciation model with constant split fraction, the Blum–François beta-splitting model, and the Sainudiin–Véber beta-splitting model. Because a parameter choice of α=0 in the Blum–François beta-splitting model corresponds to the Yule model, we have also found a new proof for the expected value of the Sackin index under the Yule model.

The new result provides a rare example of an exact mathematical result for a tree balance index under a distribution other than the Yule and Uniform models. Many exact results about tree shape are available for the Yule and Uniform models (Fischer et al. 2023). Some approximate and limiting results are also available for the Sackin and other indices in other probability models (e.g. Aldous 1996; Ford 2006; Fuchs and Paningbatan 2020; Kaur et al. 2023; Aldous and Pittel 2025). However, few exact results about tree balance indices are available in models beyond the Yule and Uniform, exceptions including the mean and variance of the rooted quartet index for the Aldous beta-splitting model and Ford’s α-model (Coronado et al. 2019), and Sackin results under uniform models on various network classes (Zhang 2022; Chang et al. 2024; Fuchs and Gittenberger 2025).

Among probability models in phylogenetics, the general biased speciation model is contained in the class of forward-time models known as *discrete-time tree-growing models without extinction* (Kersting et al., 2025, Table 4). Such models are interpretable as speciation models because they generate trees by producing new species at the leaves of a current tree, representing a “forward-time” process. Their defining feature is a rule that takes the speciation rate *p* of a parent node and assigns speciation rates $p_L$ and $p_R$ to its left and right descendants. The general biased speciation model sets $p_L = p(1-X)$ and $p_R = pX$ for a split fraction *X*, but many other rules are possible (Kersting et al., 2025, Table 4). It will be useful to explore the Sackin index in broader classes of models beyond the general biased speciation model.

The proofs of many results about tree statistics utilize recursive techniques based on splitting the tree into subtrees from the root node (Blum et al. 2006; Matsen 2007; Chang and Fuchs 2009; Holmgren and Janson 2015; Fuchs 2025). Our proof for the expectation of the Sackin index under the general biased speciation model instead employs a less frequently used form of recursion on a single time step in the evolutionary model (Wu and Choi 2016; Choi et al. 2020). The recursive technique we apply (Lemma A7) is a general technique of using recursion to find the expected value of terms in a random sequence. The property of our specific situation that made this calculation feasible is that in a single time step, changes in the Sackin index occur in the new leaves of a tree after a split, and changes in split probabilities in the model also only occur in these leaves. As a result, to generate the required recursive equations, it is straightforward to account for the way the Sackin index and the split probabilities of each leaf change in a single time step. It may be possible to use similar reasoning for other calculations that can be described in terms of change in single time steps: for example, to calculate properties of other tree statistics that are defined as functions of the leaf depths, such as the $B_2$ index (Fischer et al., 2023, ch. 11) and the variance of leaf depths (Fischer et al., 2023, ch. 7), as well as to obtain higher moments of the Sackin index.

For the general biased speciation model with constant split fraction, we observe that the expected value of the Sackin index F(n,λ) grows with $\Theta (n^2)$ for λ=0 (Eq. 10). Future work could investigate the large-*n* asymptotics of the mean Sackin index with particular parameter values in Eqs. 9, 11, and 21. Such results would clarify if, for each case of the model, the Sackin index behaves asymptotically like $\Theta (n^2)$ as in the maximal value (Eq. 3), $\Theta (n^{3/2})$ as in the Uniform model (Blum et al. 2006), $\Theta (n\log {n})$ as in the minimal value (Eq. 4)—also achieved in the Yule model (Eq. 5), equivalently to the Blum–François model with parameter α=0 (Eq. 16)—or some other rate.

A goal for phylogenetic models is to facilitate comparisons with data. By extending the evaluation of tree balance beyond classic phylogenetic models, our results provide new theoretical insights for comparing observed tree balance values to a biased speciation scenario to further understand the processes that generate the observed patterns.

## Data Availability

The paper has no associated data.

## Proof of Theorem 2

The general strategy of the proof of Theorem 2 is to calculate the expected change in the Sackin index *S*(*T*) in a single time step in the general biased speciation model. For an unlabeled history $\{T_1, T_2, \ldots , T_n\}$ generated by the general biased speciation model, we study conditional expectations $\mathbb {E}\big [S(T_{k+1}) \, | \, T_k\big ]$ and recursively use them to find $\mathbb {E}\big [S(T_n)\big ]$. The central challenge is that $\mathbb {E}\big [S(T_{k+1}) \, | \, T_k\big ]$ depends not only on the Sackin index at previous times in the model, but also on other properties of the tree, including split probabilities at the leaves. We therefore define auxiliary statistics that incorporate information about these split probabilities and find a system of equations for the Sackin index and the auxiliary statistics. Using a technical lemma (Lemma A7), we demonstrate a general method that solves the system, completing the proof of Theorem 2 to find the expectation of the Sackin index under the general biased speciation model.

The organization of Appendix A is as follows. First, we introduce the relevant probability background and notation (Sect. A.1). We then prove a series of recursive relationships for the Sackin index and the auxiliary statistics (Sect. A.2). We prove our technical lemma (Sect. A.3). Finally, we finish the calculation to prove the theorem (Sect. A.4).

### Probability background

We present elementary background and notation for probability spaces as used in this appendix; see e.g. Durrett (Durrett, 2019, Chapters 1 & 4). A *probability space* is a triple $(\Omega , \mathcal {F}, \mathbb {P})$, where Ω is the set of outcomes, $\mathcal {F}$ is a σ-algebra of events, and $\mathbb {P}: \mathcal {F} \rightarrow [0,1]$ is a function assigning probability to each event.

Fix n≥1. The random process that generates an unlabeled history $\{T_1, T_2, \ldots , T_n\}$ under the general biased speciation model corresponds to a probability space, where the underlying sample space Ω is the set of all unlabeled histories of size *n* and the event space $\mathcal {F}_n$ is the σ-algebra of all subsets of Ω. The *filtration* $(\mathcal {F}_k)_{1 \le k \le n}$ is a nested sequence of σ-algebras $\mathcal {F}_1 \subset \mathcal {F}_2 \subset \cdots \subset \mathcal {F}_n$, where $\mathcal {F}_k$ is the event space that is defined up to time *k* for the unlabeled history. Informally, $\mathcal {F}_k$ is the information about the unlabeled history up to time *k*, meaning that all properties of the sequence $\{T_1, T_2, \ldots , T_k\}$ are known (including the tree topology and the split probabilities of the leaves $p_v$), and that future trees in the history are unknown. The probability law $\mathbb {P}$ is the probability of observing a particular outcome under the model. The random split fraction $X_k \sim X$ is sampled at time *k* in the model and is independent of $\mathcal {F}_k$, but contained in $\mathcal {F}_{k+1}$.

A *tree shape statistic* is a function $B: \mathcal {T} \mapsto \mathbb {R}$. Under the general biased speciation model, a tree shape statistic *B* follows a discrete-time stochastic process $\big ( B(T_k) \big )_{1 \le k \le n}$, where $\{T_1, T_2, \ldots , T_n\}$ is a random unlabeled history generated by the model. Each random variable $B(T_k)$ is $\mathcal {F}_k$-*measurable*. Informally, $B(T_k)$ can be computed with the information in the history up to time *k*. This property means that the stochastic process is *adapted to the filtration*. The concept of being adapted to the filtration naturally extends to a vector of random variables $\big (\vec {\mathcal {B}}_k \big )_{1 \le k \le n}$, where $\vec {\mathcal {B}}_k = \big (B_1(T_k), B_2(T_k), \ldots , B_r(T_k) \big )^t$.

### Recursive system of equations for the Sackin index

Let $T \in \mathcal {T}$ be a tree generated under the general biased speciation model. For a leaf $v \in V_L(T)$, recall that $\delta _T(v)$ denotes the path length from *v* to the root, and $p_v$ denotes the probability that *v* is the next to split under the model. Recall that the Sackin index *S*(*T*) is defined by Eq. 1.

We define the following sums of a similar form. These quantities serve as auxiliary statistics that measure properties of the split probabilities of the leaves in the general biased speciation model:

A1 $$\begin{aligned} S_p(T) = \sum _{v \in V_L(T)} p_v \, \delta _T(v), \end{aligned}$$

and for j≥2,

A2 $$\begin{aligned} P_j(T) = \sum _{v \in V_L(T)} p_v^j. \end{aligned}$$

In the propositions that follow, we calculate recursive equations of the form $\mathbb {E}\big [f(T_{k+1}) \, | \, \mathcal {F}_k\big ]$, where *f*(*T*) is $S(T), S_p(T)$, or $P_j(T)$. In particular, we will express this conditional expectation as a linear combination of these same tree statistics applied to the tree $T_k$.

The following two propositions calculate $\mathbb {E}\big [S(T_{k+1}) \ | \ \mathcal {F}_k \big ]$.

#### Proposition A1

Let $\{T_1, T_2, \ldots , T_n\}$ be a random sequence of trees generated under the general biased speciation model with split fraction *X*. Let $1 \le k \le n-1$. For a vertex $w \in V_L(T_k)$, let $E_w$ denote the event that *w* is the vertex chosen to split at time *k*. Then

(i) $$S(T_{k+1}) \, | \, \mathcal {F}_k, E_w = S(T_k) + 2 + \delta _{T_k}(w)$$.

(ii) $$\mathbb {E} \big [S(T_{k+1}) \, | \, \mathcal {F}_k, E_w \big ] = S(T_k) + 2 + \delta _{T_k}(w).$$

#### Proof

(i) Conditioning on $\mathcal {F}_k$, we assume $T_{k}$ is known. Suppose $w \in V_L(T_{k})$ is the leaf that branches at time *k*, so that we condition on $E_w$. If $w_1,w_2$ are the two descendants of *w*, then the leaf set $V_L(T_{k+1})$ is

A3 $$\begin{aligned} V_L(T_{k+1}) = \big (V_L(T_{k}) \setminus \{w\}\big ) \sqcup \{w_1, w_2\}. \end{aligned}$$

The Sackin index $S(T_{k+1})$ conditional on $\mathcal {F}_{k}$ and $E_w$ is

$$\begin{aligned} S(T_{k+1}) \, | \, \mathcal {F}_k, E_w&= \sum _{v \in V_L(T_{k+1})} \delta _{T_{k+1}}(v) \\&=\bigg [ \sum _{\begin{array}{c} v \in V_L(T_k) \\ v \ne w \end{array}} \delta _{T_{k+1}}(v) \bigg ] + 2\big [1 + \delta _{T_k}(w)\big ] \\&=\bigg [\sum _{v \in V_L(T_k)} \delta _{T_k}(v)\bigg ] + 2 + \delta _{T_k}(w) \\&= S(T_k) + 2 + \delta _{T_k}(w), \end{aligned}$$

where in the third step we used the fact that $\delta _{T_k}(v) = \delta _{T_{k+1}}(v)$ for vertices v≠w.

(ii) This statement immediately follows by taking expectations and noting that the right hand side is deterministic conditional on $\mathcal {F}_k, E_w$. □

We immediately apply this proposition to prove the following result.

#### Proposition A2

Let $\{T_1, T_2, \ldots , T_n\}$ be a random sequence of trees generated under the general biased speciation model with split fraction *X*. Let $1 \le k \le n-1$. Then

$$\begin{aligned} \mathbb {E}\big [S(T_{k+1}) \, | \, \mathcal {F}_k \big ] = S(T_k) + 2 + S_p(T_k). \end{aligned}$$

#### Proof

By the law of total probability and Proposition A1,

$$\begin{aligned} \mathbb {E}\big [S(T_{k+1}) \, | \, \mathcal {F}_k \big ]&= \sum _{w \in V_L(T_k)} \mathbb {P}[E_w] \, \mathbb {E}\big [ S(T_{k+1}) \, | \, \mathcal {F}_k, E_w \big ] \\&= \sum _{w \in V_L(T_k)} p_w \big [S(T_k) + 2 + \delta _{T_k}(w) \big ] \\&= S(T_k) + 2 + \sum _{w \in V_L(T_k)} p_w \, \delta _{T_k}(w) \\&= S(T_k) + 2 + S_p(T_k), \end{aligned}$$

using $\sum _{v \in V_L(T)} p_v = 1$ in the third step, and the definition of $S_p(T)$ from Eq. A1 in the final step. □

If the relationship in Proposition A2 had the form $\mathbb {E}\big [S(T_{k+1}) \ | \ \mathcal {F}_k\big ] = a_k + b_k S(T_k)$, then it would be possible to take the expectation of both sides and solve a recursive equation to find $\mathbb {E}\big [S(T_n)\big ]$. However, the equation includes the auxiliary statistic $S_p(T_k)$ that has unknown expectation. We next examine the conditional expectation of $S_p(T)$.

#### Proposition A3

Let $\{T_1, T_2, \ldots , T_n\}$ be a random sequence of trees generated under the general biased speciation model with split fraction *X*. Let $1 \le k \le n-1$. For a vertex $w \in V_L(T_k)$, let $E_w$ denote the event that *w* is the vertex chosen to split at time *k*. Then

$$\begin{aligned} \mathbb {E}\big [S_p(T_{k+1}) \, | \, \mathcal {F}_k, E_w\big ] = S_p(T_k) + p_w. \end{aligned}$$

#### Proof

Conditioning on $\mathcal {F}_k$, $T_{k}$ is known. Suppose that $w \in V_L(T_{k})$ is the leaf that branches at time *k*. Let $w_1, w_2$ be the two descendants of *w* in $T_{k+1}$. By Eqs. A1 and A3,

A4 $$\begin{aligned} \mathbb {E}\big [S_p(T_{k+1}) \, | \, \mathcal {F}_k, E_w\big ]&= \mathbb {E} \bigg [\sum _{v \in V_L(T_{k+1})} p_v \,\delta _{T_{k+1}}(v) \, \bigg | \, \mathcal {F}_k, E_w \bigg ] \nonumber \\&= \bigg [\sum _{\begin{array}{c} v \in V_L(T_k) \\ v \ne w \end{array}} \mathbb {E}\big [p_v \, \delta _{T_{k+1}}(v) \, | \, \mathcal {F}_k, E_w] \bigg ] \nonumber \\&\qquad + \mathbb {E} \bigg [p_{w_1} \, \delta _{T_{k+1}}(w_1)+ p_{w_2} \, \delta _{T_{k+1}}(w_2) \, \bigg | \, \mathcal {F}_k, E_w \bigg ] \nonumber \\&= \bigg [\sum _{\begin{array}{c} v \in V_L(T_k) \\ v \ne w \end{array}} p_v \, \delta _{T_k}(v) \bigg ]\nonumber \\&\qquad + \mathbb {E} \bigg [p_{w_1} \, \delta _{T_{k+1}}(w_1) + p_{w_2} \, \delta _{T_{k+1}}(w_2) \, \bigg | \, \mathcal {F}_k, E_w \bigg ]. \end{aligned}$$

Here, we have used the fact that $p_v$ and $\delta _{T}(v)$ remain unchanged for vertices that did not split at time *k*. For the descendants of *w*, we have $\delta _{T_{k+1}}(w_1) = \delta _{T_{k+1}}(w_2) = 1 + \delta _{T_{k}}(w)$. Additionally, in the general biased speciation model with split fraction *X*, $p_{w_1} = (1 - X_{k+1}) \, p_w$ and $p_{w_2} = X_{k+1} \, p_w$, where $X_{k+1} \sim X$ is independent of $\mathcal {F}_k$. Hence,

A5 $$\begin{aligned}&\mathbb {E} \bigg [p_{w_1} \, \delta _{T_{k+1}}(w_1) + p_{w_2} \, \delta _{T_{k+1}}(w_2) \, \bigg | \, \mathcal {F}_k, E_w \bigg ] \nonumber \\&\quad = \mathbb {E} \bigg [p_w \, (1 - X_{k+1}) \, \big [1 + \delta _{T_k}(w)\big ] + p_w \, X_{k+1} \, \big [1 + \delta _{T_k}(w)\big ] \, \bigg | \, \mathcal {F}_k, E_w \bigg ] \nonumber \\&\quad = \big [1 + \delta _{T_k}(w)\big ] \, p_w \, \mathbb {E}\big [(1 - X_{k+1}) + X_{k+1} \, | \, \mathcal {F}_k, E_w \big ] \nonumber \\&\quad = \big [1 + \delta _{T_k}(w) \big ] \, p_w. \end{aligned}$$

We combine Eqs. A4 and A5:

$$\begin{aligned} \mathbb {E}\big [S_p(T_{k+1}) \, | \, \mathcal {F}_k, E_w\big ]&= \bigg [\sum _{\begin{array}{c} v \in V_L(T_k) \\ v \ne w \end{array}} p_v \delta _{T_k}(v) \bigg ] + \big [1 + \delta _{T_k}(w)\big ] \, p_w = S_p(T_{k}) + p_w, \end{aligned}$$

and obtain the desired result. □

We immediately apply this proposition to prove the next proposition.

#### Proposition A4

Let $\{T_1, T_2, \ldots , T_n\}$ be a random sequence of trees generated under the general biased speciation model with split fraction *X*. Let $1 \le k \le n-1$. Then

$$\begin{aligned} \mathbb {E}\big [S_p(T_{k+1}) \, | \, \mathcal {F}_k \big ] = S_p(T_k) + P_2(T_k). \end{aligned}$$

#### Proof

By the law of total probability and Proposition A3,

$$\begin{aligned} \mathbb {E}\big [S_p(T_{k+1}) \, | \, \mathcal {F}_k \big ]&= \sum _{w \in V_L(T_k)} \mathbb {P}[E_w] \, \mathbb {E}\big [ S_p(T_{k+1}) \, | \, \mathcal {F}_k, E_w \big ] \\&= \sum _{w \in V_L(T_k)} p_w \big [S_p(T_k) + p_w \big ] = S_p(T_k) + P_2(T_k), \end{aligned}$$

where we have used the fact that $\sum _{v \in V_L(T)} p_v = 1$ and the definition of $P_2(T)$ from Eq. A2. □

Proposition A4 shows that the equation for the expectation of $S_p(T_{k+1})$ in terms of $S_p(T_k)$ involves a new quantity $P_2(T_k)$ that has unknown expectation. The following two propositions calculate conditional expectations of $P_j(T_{k+1})$.

#### Proposition A5

Let $\{T_1, T_2, \ldots , T_n\}$ be a random sequence of trees generated under the general biased speciation model with split fraction *X*. Let $1 \le k \le n-1$. For a vertex $w \in V_L(T_k)$, let $E_w$ denote the event that *w* is the vertex chosen to split at time *k*. Let j≥2. Then

$$\begin{aligned} \mathbb {E}\big [P_j(T_{k+1}) \, | \, \mathcal {F}_k, E_w\big ] = P_j(T_k) + e_j \, p_w^j, \end{aligned}$$

where $$e_j = \mathbb {E}\big [X^j \big ] + \mathbb {E}\big [(1-X)^j \big ] - 1$$.

#### Proof

Conditioning on $\mathcal {F}_k$, $T_{k}$ is known. Suppose that $w \in V_L(T_{k})$ is the leaf that branches at time *k*. Let $w_1, w_2$ be the two descendant leaves of *w* in $T_{k+1}$. By Eqs. A2 and A3, using the linearity of the expectation,

A6 $$\begin{aligned} \mathbb {E}\big [P_j(T_{k+1}) \, | \, \mathcal {F}_k, E_w\big ]&= \mathbb {E} \bigg [\sum _{v \in V_L(T_{k+1})} p_v^j \, \bigg | \, \mathcal {F}_k, E_w \bigg ] \nonumber \\&= \bigg [\sum _{\begin{array}{c} v \in V_L(T_k) \\ v \ne w \end{array}} \mathbb {E}\big [p_v^j \, | \, \mathcal {F}_k, E_w] \bigg ] + \mathbb {E} \big [p_{w_1}^j + p_{w_2}^j \, | \, \mathcal {F}_k, E_w \big ], \nonumber \\&= \bigg [\sum _{\begin{array}{c} v \in V_L(T_k) \\ v \ne w \end{array}} p_v^j \bigg ] + \mathbb {E} \big [p_{w_1}^j + p_{w_2}^j \, | \, \mathcal {F}_k, E_w \big ]. \end{aligned}$$

Recall that $p_v$ remains unchanged for all vertices that do not split at time *k*. Under the general biased speciation model, $p_{w_1} = (1 - X_{k+1}) \, p_w$ and $p_{w_2} = X_{k+1} \, p_w$, where $X_{k+1} \sim X$ is independent of $\mathcal {F}_k$ and $E_w$, so

A7 $$\begin{aligned} \mathbb {E} \big [p_{w_1}^j + p_{w_2}^j \, | \, \mathcal {F}_k, E_w \big ]&= \mathbb {E} \big [p_w^j (1 - X_{k+1})^j + p_w^j X_{k+1}^j \, | \, \mathcal {F}_k, E_w \big ] \nonumber \\&= p_w^j \, \mathbb {E}\big [(1 - X_{k+1})^j + X_{k+1}^j \big ]. \end{aligned}$$

Combining Eqs. A6 and A7 gives

$$\begin{aligned} \mathbb {E}\big [P_j(T_{k+1}) \, | \, \mathcal {F}_k, E_w\big ]&= \bigg [\sum _{\begin{array}{c} v \in V_L(T_k) \\ v \ne w \end{array}} p_v^j \bigg ] + p_w^j \, \mathbb {E}\big [(1 - X_{k+1})^j + X_{k+1}^j \big ] \\&= P_j(T_{k}) - p_w^j + p_w^j \, \big (\mathbb {E}\big [ (1 - X)^j\big ] + \mathbb {E}\big [X^j\big ]\big ) \\&= P_j(T_k) + e_j \, p_w^j, \end{aligned}$$

where the last step applies the definition of $e_j$. □

We immediately apply this proposition to prove the following result.

#### Proposition A6

Let $\{T_1, T_2, \ldots , T_n\}$ be a random sequence of trees generated under the general biased speciation model with split fraction *X*. Let $1 \le k \le n-1$, and let j≥2. Then

$$\begin{aligned} \mathbb {E}\big [P_j(T_{k+1}) \, | \, \mathcal {F}_k \big ] = P_j(T_{k}) + e_j \, P_{j+1}(T_{k}), \end{aligned}$$

where $$e_j = \mathbb {E}\big [X^j \big ] + \mathbb {E}\big [(1-X)^j \big ] - 1$$.

#### Proof

By the law of total probability and Proposition A5,

$$\begin{aligned} \mathbb {E}\big [P_j(T_{k+1}) \, | \, \mathcal {F}_k \big ]&= \sum _{w \in V_L(T_k)} \mathbb {P}[E_w] \, \mathbb {E}\big [ P_j(T_{k+1}) \, | \, \mathcal {F}_k, E_w \big ] \\&= \sum _{w \in V_L(T_k)} p_w \big [P_j(T_k) + e_j \, p_w^j \big ] \\&= P_j(T_k) + e_j \, P_{j+1}(T_k). \\ \end{aligned}$$

□

We have demonstrated that the conditional expectation for the auxiliary statistic $P_j(T_{k+1})$ can be written recursively in terms of $P_{j+1}(T_k)$. As a result, although the recursion increments the exponent *j* by 1, its structure does enable us to recursively obtain the expectation of $S(T_{k+1})$ in terms of quantities computed for $T_k$. We combine Propositions A2, A4, and A6 into one equation as an (infinite-dimensional) array, in which, outside of the tridiagonal entries shown, all entries equal zero:

A8 $$\begin{aligned} \mathbb {E}\left\{ \left[ \begin{array}{c} 1 \\ S(T_{k+1}) \\ S_p(T_{k+1}) \\ P_2(T_{k+1}) \\ P_3(T_{k+1}) \\ \vdots \\ P_j(T_{k+1}) \\ \vdots \end{array}\right] \bigg | \mathcal {F}_{k}\right\} = \left[ \begin{array}{ccccccccc} 1 & \quad & \quad & \quad & \quad & \quad & \quad & \quad \\ 2 & \quad 1 & \quad 1 & \quad & \quad & \quad & \quad & \quad \\ & \quad & \quad 1 & \quad 1 & \quad & \quad & \quad \\ & \quad & \quad & \quad 1 & \quad e_2 & \quad & \quad & \quad \\ & \quad & \quad & \quad & \quad 1 & \quad e_3 & \quad & \quad \\ & \quad & \quad & \quad & \quad & \quad \ddots & \quad \ddots & \quad \\ & \quad & \quad & \quad & \quad & \quad & \quad 1 & \quad e_j & \quad \\ & \quad & \quad & \quad & \quad & \quad & \quad & \quad \ddots & \quad \ddots \end{array}\right] \left[ \begin{array}{c} 1 \\ S(T_{k}) \\ S_p(T_{k}) \\ P_2(T_{k}) \\ P_3(T_{k}) \\ \vdots \\ P_j(T_{k}) \\ \vdots \end{array}\right] . \end{aligned}$$

The starting vector $\vec {A}_1$ consists of constants:

A9 $$\begin{aligned} \vec {A}_1 = \left[ \begin{array}{c} 1 \\ S(T_{1}) \\ S_p(T_{1}) \\ P_2(T_{1}) \\ P_3(T_{1}) \\ \vdots \\ P_j(T_{1}) \\ \vdots \end{array}\right] =\left[ \begin{array}{c} 1 \\ 0 \\ 0 \\ 1 \\ 1 \\ \vdots \\ 1 \\ \vdots \end{array}\right] . \end{aligned}$$

### A technical lemma

To complete the derivation of $\mathbb {E}[S(T_n)]$, we need one additional lemma. We write this lemma in a general form suited to a class of recursive computations of expectations.

The subscript n≥1 refers to a time variable. We consider a vector of random variables $A_n^{(i)}$; the superscript (*i*), with i≥1, provides the index of the *i*th of a set of random variables that are considered at a given point in time. The lemma computes the conditional expectation of random variable $A_{n+1}^{(i)}$ in terms of the various random variables $A_{n}^{(j)}$ at time *n*, given certain assumptions about the linear dependencies of the random variables at time n+1 in terms of those at time *n*.

We require two additional quantities for the statement of the lemma. The first is *r*, a quantity that describes which random variables contribute linearly to the conditional expectation; it counts the number of diagonals that lie immediately above the main diagonal of the matrix in Eq. A8 and that contain non-zero entries. The second is *m*, which describes the total number of random variables that are considered in the lemma when selecting a finite submatrix of the infinite matrix in Eq. A8.

#### Lemma A7

Let $(\Omega ,\mathcal {F}, \mathbb {P})$ be a probability space with a filtration $(\mathcal {F}_n)_{n \ge 1}$. For each integer i≥1, let $\big ( A_n^{(i)} \big )_{n\ge 1}$ be a stochastic process that is adapted to the filtration. Suppose there exists an integer r≥0 such that for all i≥1 and n≥1,

A10 $$\begin{aligned} \mathbb {E}\big [A^{(i)}_{n+1} \, | \, \mathcal {F}_{n} \big ] = \sum _{j =1}^{i + r} c_{i,j} \, A^{(j)}_{n}, \end{aligned}$$

for coefficients $$c_{i,j} \in \mathbb {R}$$.

Suppose that for any integer m≥1, $\textbf{C}_m$ is the m×m matrix whose (*i*, *j*) entry is $c_{i,j}$, where by convention $c_{i,j} = 0$ for all j>i+r. Let $\vec {A}_m$ be a column vector of length *m* with entry *i* equal to $\mathbb {E}[A^{(i)}_1]$.

Then for a fixed i≥1, fixed n≥1, and all m≥i+(n-1)r, the expectation $\mathbb {E}[A_n^{(i)}]$ is equal to the *i*th element of the vector $(\textbf{C}_{m})^{n-1} \vec {A}_m$, or

A11 $$\begin{aligned} \mathbb {E}\big [ A_n^{(i)} \big ] = \big ((\textbf{C}_{m})^{n-1} \vec {A}_m \big )_i. \end{aligned}$$

#### Proof

We proceed by induction on *n*. The base case of n=1 follows immediately from the definition of $\vec {A}_m$, which has an *i*th element as long as m≥i.

For the inductive step, let n≥1 and suppose that the hypotheses of the lemma and the result in Eq. A11 hold through time *n* for all i≥1. We show that the result holds at time n+1 for all i≥1. Fix i≥1. By the law of iterated conditional expectation and Eq. A10,

A12 $$\begin{aligned} \mathbb {E}\big [ A_{n+1}^{(i)}\big ] = \mathbb {E}\big [ \mathbb {E}[A_{n+1}^{(i)} \, | \, \mathcal {F}_{n}]\big ] = \sum _{j=1}^{i + r} c_{i,j} \mathbb {E}\big [ A_{n}^{(j)}\big ], \end{aligned}$$

where we have also used the linearity of expectation.

Let *m* be an integer satisfying m≥i+nr, so that m≥j+(n-1)r for each *j* with $1 \le j \le i + r$. Therefore, we can apply the inductive hypothesis to $A^{(j)}_{n}$:

A13 $$\begin{aligned} \mathbb {E}\big [ A_{n}^{(j)}\big ] = \big ((\textbf{C}_m)^{n-1} \vec {A}_m \big )_j. \end{aligned}$$

Combining Eqs. A12 and A13 then gives

$$\begin{aligned} \mathbb {E}\big [ A_{n+1}^{(i)}\big ]&= \sum _{j=1}^{i + r} c_{i,j} \big ((\textbf{C}_m)^{n-1} \vec {A}_m \big )_j \\&= \sum _{j=1}^{m} c_{i,j} \big ((\textbf{C}_m)^{n-1} \vec {A}_m \big )_j \\&= \big ((\textbf{C}_m)^{n} \vec {A}_m \big )_i, \end{aligned}$$

where we used the fact that $c_{i,j} = 0$ for j>i+r, and the definition of the matrix product. □

#### Remark A9

Lemma A7 can be straightforwardly generalized to a scenario in which the relationship in Eq. A10 has coefficients that depend on *n*. In this scenario, we label the coefficients $c_{i,j; n}$ and construct matrices $\textbf{C}_{m; n}$. The matrix power $(\textbf{C}_m)^{n-1}$ in Eq. A11 is replaced with $\textbf{C}_{m; n-1}\textbf{C}_{m;n-2}\cdots \textbf{C}_{m,1}$, the matrix product of n-1 time-dependent coefficient matrices. The proof is otherwise the same.

### Proof of the main theorem

To prove Theorem 2, we apply Lemma A7 to the results obtained in Sect. A.2. Fix n≥1. In the language of Lemma A7, we have a sequence of stochastic processes:

$$\begin{aligned} A^{(1)}&= \big (1\big )_{1 \le k \le n}, \\ A^{(2)}&= \big ( S(T_k) \big )_{1 \le k \le n}, \\ A^{(3)}&= \big ( S_p(T_k) \big )_{1 \le k \le n}, \\ A^{(i)}&= \big ( P_{i - 2}(T_k) \big )_{1 \le k \le n} {\text { for }} i \ge 4. \end{aligned}$$

For all *i* and *k*, i≥1 and $1 \le k \le n-1$,

$$\begin{aligned} \mathbb {E}\big [A^{(i)}_{k+1} \, | \, \mathcal {F}_k\big ] = \sum _{j=1}^{i + 1} c_{i,j} A^{(j)}_k, \end{aligned}$$

where the coefficients $c_{i,j}$ are precisely the entries (*i*, *j*) in the infinite array of Eq. A8. Therefore, the conditions of Lemma A7 are satisfied with r=1.

We now apply Lemma A7 to the Sackin index, corresponding to i=2. Fix n≥1, and let m=i+(n-1)r=2+(n-1)1=n+1. By the lemma,

A14 $$\begin{aligned} \mathbb {E}\big [ S(T_n) \big ] = \mathbb {E} \big [ A^{(2)}_{n} \big ] = \big ((\textbf{C}_{n+1})^{n-1} \vec {A}_{n+1} \big )_2, \end{aligned}$$

where $\textbf{C}_{n+1}$ is the matrix consisting of the first n+1 rows and columns of the infinite array in Eq. A8, and $\vec {A}_{n+1}$ is the vector of the first n+1 elements in Eq. A9.

To calculate $\big ((\textbf{C}_{n + 1})^{n-1} \vec {A}_{n+1} \big )_2$, observe that we can decompose $\textbf{C}_{n+1}$ as

$$\begin{aligned} \textbf{C}_{n+1}= \left[ \begin{array}{ccccccccc} 1 & \quad 0 & \quad & \quad & \quad & \quad & \quad & \quad \\ 2 & \quad 1 & \quad 1 & \quad & \quad & \quad & \quad & \quad \\ & \quad & \quad 1 & \quad 1 & \quad & \quad & \quad \\ & \quad & \quad & \quad 1 & \quad e_2 & \quad & \quad & \quad \\ & \quad & \quad & \quad & \quad 1 & \quad e_3 & \quad & \quad \\ & \quad & \quad & \quad & \quad & \quad \ddots & \quad \ddots \\ & \quad & \quad & \quad & \quad & \quad & \quad 1 & \quad e_{n-2} \\ & \quad & \quad & \quad & \quad & \quad & \quad & \quad 1 \end{array}\right] = \textbf{I} + \textbf{B}, \end{aligned}$$

where $\textbf{I}$ is the (n+1)×(n+1) identity matrix, and

$$\begin{aligned} \textbf{B} = \left[ \begin{array}{ccccccccc} 0 & \quad 0 & \quad & \quad & \quad & \quad & \quad & \quad \\ 2 & \quad 0 & \quad 1 & \quad & \quad & \quad & \quad & \quad \\ & \quad & \quad 0 & \quad 1 & \quad & \quad & \quad \\ & \quad & \quad & \quad 0 & \quad e_2 & \quad & \quad & \quad \\ & \quad & \quad & \quad & \quad 0 & \quad e_3 & \quad & \quad \\ & \quad & \quad & \quad & \quad & \quad \ddots & \quad \ddots \\ & \quad & \quad & \quad & \quad & \quad & \quad 0 & \quad e_{n-2} \\ & \quad & \quad & \quad & \quad & \quad & \quad & \quad 0 \end{array}\right] . \end{aligned}$$

Matrices $\textbf{I}$ and $\textbf{B}$ commute, $\textbf{IB}=\textbf{BI}=\textbf{B}$. Therefore, by the binomial theorem,

A15 $$\begin{aligned} (\textbf{C}_{n+1})^{n-1} = \big (\textbf{I} + \textbf{B} \big )^{n-1} = \sum _{k=0}^{n-1} \left( {\begin{array}{c}n-1\\ k\end{array}}\right) \textbf{B}^k, \end{aligned}$$

where, by convention, $\textbf{B}^0 = \textbf{I}$.

Next, we observe that for $2 \le k \le n-1$, the matrix $\textbf{B}^k$ is 0 except for the diagonal *k* steps above the main diagonal; for example

$$\begin{aligned} \textbf{B}^2 = \left[ \begin{array}{cccccccccc} 0 & \quad 0 & \quad 0 & \quad & \quad & \quad & \quad & \quad & \quad \\ & \quad 0 & \quad 0 & \quad 1& \quad & \quad & \quad & \quad & \quad \\ & \quad & \quad 0 & \quad 0 & \quad e_2 & \quad & \quad & \quad \\ & \quad & \quad & \quad 0 & \quad 0 & \quad e_2e_3 & \quad & \quad & \quad \\ & \quad & \quad & \quad & \quad 0 & \quad 0 & \quad e_3e_4 & \quad & \quad \\ & \quad & \quad & \quad & \quad & \quad & \quad & \quad & \quad \\ & \quad & \quad & \quad & \quad & \quad \ddots & \quad \ddots & \quad \ddots & \quad \\ & \quad & \quad & \quad & \quad & \quad & \quad & \quad & \quad \\ & \quad & \quad & \quad & \quad & \quad & \quad 0 & \quad 0 & \quad e_{n-3}e_{n-2}\\ & \quad & \quad & \quad & \quad & \quad & \quad & \quad 0 & \quad 0\\ & \quad & \quad & \quad & \quad & \quad & \quad & \quad & \quad 0 \end{array}\right] . \end{aligned}$$

For a matrix $\textbf{B}$, we denote the entries in this nonzero diagonal, which has length n+1-k, as $\text {Diag}_k(\textbf{A}) = \big (B_{i, i+k} \big )_{i = 1}^{n+1-k}$. We calculate these upper diagonal entries as

$$\begin{aligned} \text {Diag}_2(\textbf{B}^{2})&= \big (0, 1, e_2, e_2e_3, e_3e_4, \ldots , e_{n-3}e_{n-2} \big ), \\ \text {Diag}_3(\textbf{B}^{3})&= \big (0, e_2, e_2e_3, e_2e_3e_4, e_3e_4e_5, \ldots , e_{n-4}e_{n-3}e_{n-2} \big ), \\ \text {Diag}_4(\textbf{B}^{4})&= \big (0, e_2e_3, e_2e_3e_4, e_2e_3e_4e_5, e_3e_4e_5e_6, \ldots , e_{n-5}e_{n-4}e_{n-3}e_{n-2} \big ), \\ \vdots \\ \text {Diag}_{n-1}(\textbf{B}^{n-1})&= \big (0, e_2e_3\cdots e_{n-2}). \end{aligned}$$

Combining these expressions with Eq. A9, we calculate that $\big (\textbf{I} \vec {A}_{n+1}\big )_2 = 0$, $\big (\textbf{B} \vec {A}_{n+1}\big )_2 = 2$, $\big (\textbf{B}^2 \vec {A}_{n+1} \big )_2 = 1$, and for $3 \le k \le n-1$, $\big (\textbf{B}^k \vec {A}_{n+1} \big )_2 = \prod _{j=2}^{k - 1} e_j$. Therefore, by Eqs. A14 and A15,

$$\begin{aligned} \mathbb {E}\big [ S(T_n) \big ]&= \bigg (\sum _{k=0}^{n-1} \left( {\begin{array}{c}n-1\\ k\end{array}}\right) \, \textbf{B}^k \, \vec {A}_{n+1} \bigg )_2 \\&= \sum _{k=0}^{n-1} \left( {\begin{array}{c}n-1\\ k\end{array}}\right) \big ( \textbf{B}^{k}\vec {A}_{n+1}\big )_2 \\&= 0 + 2 \left( {\begin{array}{c}n-1\\ 1\end{array}}\right) + 1 \left( {\begin{array}{c}n-1\\ 2\end{array}}\right) + \sum _{k=3}^{n-1}\left( {\begin{array}{c}n-1\\ k\end{array}}\right) \prod _{j=2}^{k-1} e_j \\&= \frac{n^2 + n - 2}{2} + \sum _{k=3}^{n-1}\left( {\begin{array}{c}n-1\\ k\end{array}}\right) \prod _{j=2}^{k-1} e_j. \end{aligned}$$

This concludes the proof of Theorem 2.

## Alternative proof of the expected value of the Sackin index under the Yule model

Blum & François showed that the biased speciation model with split fraction $X \sim \text {Beta}(1, 1)$, a $\text {Uniform}([0,1])$ random variable, generates the same distribution of trees of size *n* as the Yule model (Blum and François 2006). We can therefore apply Theorem 2 in the special case of the general biased speciation model given by Eq. 11 to provide an alternative proof of the expectation of the Sackin index under the Yule model.

We require two binomial identities. The first is the alternating binomial identity obtained from the binomial expansion of $(-1+1)^n$:

B1 $$\begin{aligned} \sum _{k=0}^n \left( {\begin{array}{c}n\\ k\end{array}}\right) (-1)^k = 0. \end{aligned}$$

The second is the harmonic identity (Graham et al., 1994, eqs. (6.74, 6.76) with x=1, y=0),

B2 $$\begin{aligned} \sum _{k=1}^n \left( {\begin{array}{c}n\\ k\end{array}}\right) \frac{(-1)^k}{k} = - H_n. \end{aligned}$$

We now state and prove the proposition.

### Proposition B1

Let $\{T_1, T_2, \ldots , T_n\}$ be a random sequence of trees generated under the Blum–François beta-splitting model with parameter α=0, the biased speciation model with split fraction X∼Beta(1,1). The expected value of the Sackin index $S(T_n)$ is equal to

$$\begin{aligned} \mathbb {E} \big [S(T_n) \big ] = 2n\big ( H_n - 1\big ), \end{aligned}$$

where $H_n = \sum _{i=1}^n \frac{1}{i}$ is the *n*th harmonic number.

### Proof

The biased speciation model with split fraction $X \sim \text {Beta}(1, 1)$ is the Blum–François beta-splitting model with parameter α=0. Substituting α=0 into Eq. 11, we get

B3 $$\begin{aligned} \mathbb {E}\big [S(T_n)\big ]&= \frac{n^2 + n - 2}{2} + \sum _{k=3}^{n-1}\left( {\begin{array}{c}n-1\\ k\end{array}}\right) \prod _{j=2}^{k-1} \bigg [2\frac{\Gamma (1 + j) \, \Gamma (2)}{ \Gamma (2 + j) \, \Gamma (1)} - 1 \bigg ]. \end{aligned}$$

Using the identity Γ(n)=(n-1)! for integers n≥1, the product simplifies to $\prod _{j=2}^{k-1} \big [2 \, j! \, 1! / [(j+1)! \, 0!] - 1 \big ] = \prod _{j=2}^{k-1} [(-1) (j-1)/(j+1)]$, a telescoping product that reduces to $(-1)^k\frac{2}{(k-1)k} = 2(-1)^k[\frac{1}{k-1} - \frac{1}{k}]$.

Equation B3 then gives

B4 $$\begin{aligned} \mathbb {E}\big [S(T_n)\big ] = \frac{n^2 + n - 2}{2} + 2 \bigg [\sum _{k=3}^{n-1}\left( {\begin{array}{c}n-1\\ k\end{array}}\right) \frac{(-1)^k}{k-1} \bigg ] - 2 \bigg [\sum _{k=3}^{n-1}\left( {\begin{array}{c}n-1\\ k\end{array}}\right) \frac{(-1)^k}{k} \bigg ]. \end{aligned}$$

The second sum in Eq. B4 can be simplified by Eq. B2:

B5 $$\begin{aligned} \sum _{k=3}^{n-1} \left( {\begin{array}{c}n-1\\ k\end{array}}\right) \frac{(-1)^k}{k}&= -H_{n-1} + \left( {\begin{array}{c}n-1\\ 1\end{array}}\right) - \frac{1}{2}\left( {\begin{array}{c}n-1\\ 2\end{array}}\right) \nonumber \\&= -H_{n-1} - \frac{n^2 - 7n + 6}{4}. \end{aligned}$$

For the first sum, we use the identity $$\left( {\begin{array}{c}n\\ k\end{array}}\right) = \frac{n}{k}\left( {\begin{array}{c}n-1\\ k-1\end{array}}\right) $$ to rearrange as

B6 $$\begin{aligned} \sum _{k=3}^{n-1} \left( {\begin{array}{c}n-1\\ k\end{array}}\right) \frac{(-1)^k}{k-1}&= \sum _{k=3}^{n-1} \left( {\begin{array}{c}n-2\\ k-1\end{array}}\right) (-1)^k \frac{n-1}{(k-1)k} \nonumber \\&= \bigg [(n-1)\sum _{k=3}^{n-1} \left( {\begin{array}{c}n-2\\ k - 1\end{array}}\right) \frac{(-1)^{k}}{k - 1} \bigg ] - \bigg [ \sum _{k=3}^{n-1} \left( {\begin{array}{c}n-2\\ k-1\end{array}}\right) (-1)^k \frac{n-1}{k}\bigg ]. \end{aligned}$$

Shifting indices and applying Eq. B2, we find that

B7 $$\begin{aligned} (n-1)\sum _{k=3}^{n-1} \left( {\begin{array}{c}n-2\\ k - 1\end{array}}\right) \frac{(-1)^{k}}{k - 1}&= -(n-1)\sum _{k=2}^{n-2} \left( {\begin{array}{c}n-2\\ k\end{array}}\right) \frac{(-1)^{k}}{k} \nonumber \\&= -(n-1) \big [-H_{n-2} + (n - 2) \big ] \nonumber \\&= (n-1) H_{n-1} - (n^2 - 3n + 3). \end{aligned}$$

By absorbing terms into the binomial coefficient and applying Eq. B1, we have

B8 $$\begin{aligned} \sum _{k=3}^{n-1} \left( {\begin{array}{c}n-2\\ k-1\end{array}}\right) (-1)^k \frac{n-1}{k}&= \sum _{k=3}^{n-1} \left( {\begin{array}{c}n-1\\ k\end{array}}\right) (-1)^k \nonumber \\&= 0 - \left( {\begin{array}{c}n - 1\\ 0\end{array}}\right) + \left( {\begin{array}{c}n-1\\ 1\end{array}}\right) - \left( {\begin{array}{c}n-1\\ 2\end{array}}\right) \nonumber \\&= \frac{-n^2 + 5n - 6}{2}. \end{aligned}$$

Substituting Eqs. B7 and B8 into Eq. B6 then gives

B9 $$\begin{aligned} \sum _{k=3}^{n-1} \left( {\begin{array}{c}n-1\\ k\end{array}}\right) \frac{(-1)^k}{k-1} = (n-1) H_{n-1} - \frac{n^2 - n}{2}. \end{aligned}$$

To conclude the proof, we substitute Eqs. B5 and Eq. B9 into Eq. B4 and simplify to find that

$$\begin{aligned} \mathbb {E}\big [S(T_n) \big ]&= \frac{n^2 + n - 2}{2} + 2\bigg [(n-1)H_{n-1} - \frac{n^2 - n}{2}\bigg ] - 2\bigg [-H_{n-1} - \frac{n^2 - 7n + 6}{4} \bigg ] \\&= 2nH_{n-1} - 2n + 2 \\&= 2n\big (H_n - 1\big ), \end{aligned}$$

as desired. □
